# Interventions to improve the implementation of evidence-based healthcare in prisons: a scoping review

**DOI:** 10.1186/s40352-022-00200-x

**Published:** 2023-01-03

**Authors:** Jenna Blackaby, Jordan Byrne, Sue Bellass, Krysia Canvin, Robbie Foy

**Affiliations:** 1 Leeds Institute of Health Science, University of Leeds, Leeds, UK; 2 Faculty of Science and Engineering, Manchester Metropolitan University, Manchester, UK; 3 Keele University, Newcastle, UK

**Keywords:** Prison healthcare, Incarceration healthcare, Quality improvement, Intervention, Evidence-based

## Abstract

**Background:**

There are challenges to delivering high quality primary care within prison settings and well-recognised gaps between evidence and practice. There is a growing body of literature evaluating interventions to implement evidence-based practice in the general population, yet the extent and rigour of such evaluations in incarcerated populations are unknown. We therefore conducted a scoping literature review to identify and describe evaluations of implementation interventions in the prison setting.

**Methods:**

We searched EMBASE, MEDLINE, CINAHL Plus, Scopus, and grey literature up to August 2021, supplemented by hand searching. Search terms included prisons, evidence-based practice, and implementation science with relevant synonyms. Two reviewers independently selected studies for inclusion. Data extraction included study populations, study design, outcomes, and author conclusions. We took a narrative approach to data synthesis. We followed Preferred Reporting Items for Systematic Reviews and Meta-Analyses (PRISMA) guidance for scoping reviews.

**Results:**

Fifteen studies reported in 17 papers comprised one randomised controlled trial, one controlled interrupted time series analysis and 13 uncontrolled before and after studies. Eight studies took place in the US and four in the UK. Ten studies evaluated combined (multifaceted) interventions, typically including education for staff or patients. Interventions most commonly targeted communicable diseases, mental health and screening uptake. Thirteen studies reported adherence to processes of care, mainly testing, prescribing and referrals. Fourteen studies concluded that interventions had positive impacts.

**Conclusions:**

There is a paucity of high-quality evidence to inform strategies to implement evidence-based health care in prisons, and an over-reliance on weak evaluation designs which may over-estimate effectiveness. Whilst most evaluations have focused on recognised priorities for the incarcerated population, relatively little attention has been paid to long-term conditions core to primary care delivery. Initiatives to close the gaps between evidence and practice in prison primary care need a stronger evidence base.

**Supplementary Information:**

The online version contains supplementary material available at 10.1186/s40352-022-00200-x.

## Background

The global incarcerated population has grown by a quarter over in the past two decades, to 11 million in 2021 (Fair & Walmsley, 2021). Multiple social and economic disadvantages contribute to a high burden of long-term conditions, communicable diseases, mental illness, and drug misuse in this population (Condon et al., 2007; Fazel & Baillargeon, 2011; Kinner & Young, 2018; Stürup-Toft et al., 2018; Toledanes et al., 2021; Wang et al., 2017). Shifting demographics towards an ageing incarcerated population are placing further demands on healthcare systems (Forsyth et al., 2017; Ministry of Justice, 2020; Wang et al., 2017).

In the last three decades evidence-based healthcare – the translation of high-quality research into clinical practice - has become internationally accepted as essential for quality improvement, yet well-recognised gaps between recommended and actual health care and associated inappropriate variations pervade different health care settings and patient populations (Brownlee et al., 2017; Glasziou et al., 2017). This may include under-treatment and failures to meet targets for long term conditions such as diabetes and hypertension or potentially inappropriate or risky treatment (Foy et al., 2016; Willis et al., 2017). Such gaps disproportionately affect marginalised or lower socio-economic status groups, such as incarcerated persons (Rich et al., 2014; Stürup-Toft et al., 2018; World Health Organisation, 2018). For example, despite reported higher rates of cardiovascular disease in incarcerated populations compared to community populations, the availability of prescription medication, exercise and low salt diets are often out of an incarcerated person’s control (Wang et al., 2017).

Evidence-based clinical guidelines are necessary but seldom sufficient alone to bring about significant improvements in health care delivery (Grimshaw et al., 2012). This challenge is heightened in custodial settings, where adherence to guideline-recommended practice is generally lower than that for the wider population in, for example, managing cardiovascular disease, epilepsy, blood-borne viruses (BBVs), mental illness and in preventing illness through cervical screening (Chan et al., 2015; Davis et al., 2018; Elwood Martin et al., 2004; Gibson & Phillips, 2016; Humphreys et al., 2015; Kinner & Young, 2018; Meine, 2018; Tittensor et al., 2008; Wang et al., 2017). This is likely due to a confluence of factors specific to the prison healthcare context. For instance, whilst most healthcare resourcing is inevitably limited, prison services and their associated healthcare provision have generally faced tighter funding constraints (Ismail, 2020; Stephenson & Bell, 2019), with understaffing and high numbers of vacant positions compromising safety and effectiveness. There are direct impacts of healthcare understaffing; for example, two thirds of prison nurses responding to a survey in the United Kingdom stated that the care they provided on their last shift was compromised and that the quality of care was poor (Royal College of Nursing, 2018). There are also impacts of prison service understaffing; for example, a recent report from the United Kingdom noted that incarcerated people missed 20-30% of medical appointments, and that this was largely attributed to the lack of prison officers to escort incarcerated people to the healthcare wing (Association of Members of Independent Monitoring Boards, 2018). This also illustrates how the wider priorities of prison regimes substantially influence healthcare delivery; the over-riding concern with security, which has no equivalent comparison with healthcare delivered in community settings, can delay access and reduce patient autonomy (Edge et al., 2020).

Challenges in the prison setting constrain healthcare quality, yet incarceration potentially presents opportunities to address health needs that may otherwise have gone unmet in community settings, such as providing vaccinations against communicable disease and enrolment into screening programmes. Charged with ‘evaluating, promoting, protecting and improving’ the health of incarcerated people (UN General Assembly, 2016 p.8), prisons should aim to provide a standard of care at least equivalent to that available in the wider community, also known as the equivalence principle. Yet, accumulating evidence and inquiries suggest equivalence is often not achieved, compounding existing health inequities (Health and Social Care Committee, 2018). Neglecting the health needs of incarcerated people has negative implications for both the individuals concerned and for society (Leaman et al., 2016). However, as broader experience with healthcare systems indicates, concerted efforts to increase the quality of care can bring wider benefits, beyond improved health outcomes for incarcerated people, such as improved staff morale or institutional reputation (Payne, 2012).

Active implementation strategies are therefore needed to close the gap between evidence and practice to improve health outcomes for this vulnerable population. There is a growing body of evidence, based on systematic reviews of rigorous experimental and quasi-experimental evaluations, summarising the effects of a range of implementation strategies (e.g., audit and feedback, education, computerised clinical decision support) on health care delivery and outcomes in the general population (Grimshaw et al., 2012; Hillman & Roueche, 2011; Jones et al., 2019). However, the applicability of such strategies to the prison context is uncertain.

Efforts to improve the implementation of clinical guidelines in prisons needs to build on an understanding of the available and context-specific evidence on the effectiveness of implementation strategies. Otherwise, resources may be wasted on ineffective strategies and new research will fail to learn from previous work (Glasziou & Chalmers, 2018). We therefore conducted a scoping review to identify and describe studies evaluating the effects of interventions to promote the uptake of evidence-based healthcare in prison settings.

## Methods

### Design

Scoping reviews offer a systematic approach to summarise evidence on broad research topics (Arksey & O’Malley, 2005). We used the PRISMA Scoping Review (PRISMA-ScR) checklist (Tricco et al., 2018) to structure and support our review (Additional file 1: Appendix 1).

### Search strategy

We searched for and included any quantitative evaluations of interventions to improve the uptake of evidence-based practice or recommended healthcare in detention settings. We placed no limits on dates and country of origin but restricted our review to English language papers. We excluded studies of transitional care between custodial institutions and the community, those covering day release or community sentences, and those researching forensic or psychiatric inpatient populations. We excluded studies largely focused on the evaluation of clinical interventions (e.g. studies assessing the effectiveness of drug or psychological therapy for depression) as these fell outside the scope of recognised implementation strategies (Grimshaw et al., 2012). These included health promotion programmes and other interventions largely targeting the incarcerated population directly. This built in a focus on systematic changes in the prison healthcare system rather than the behaviour of incarcerated persons. However, we included evaluations including patient-mediated interventions, aimed at changing the performance of healthcare professionals through interactions with patients, or through information provided by or to patients (Fønhus et al., 2018). We excluded qualitative studies as our focus was on effectiveness evaluations but included the quantitative results from mixed-method evaluations.

Our search was focussed around three key concepts: prisons, evidence-based practice, and implementation science. Our search included synonyms of these terms, which were combined with Boolean operators. We consulted an academic librarian to determine the most relevant databases and inform our search strategy. One author (JB) then searched Medline, EMBASE, CINAHL, Scopus, and Web of Science for grey literature, searching up to August 2021 (Additional file 2: Appendix 2). The earliest dated paper for title screening was from 1978. Two reviewers (JB and JBl) checked references of all retrieved full-text papers. One reviewer (JB) hand searched two key journals (*International Journal of Prisoner Healthcare* and *Journal of Correctional Healthcare)*. During the screening process, two authors were contacted via email to request final studies from published study protocols with one response received (Almost et al., 2019). All results and responses were downloaded and imported into Endnote X9 and duplicates removed.

### Selection of literature

Two reviewers independently screened all retrieved titles (JB and Shruti Chawla, a medical student) and abstracts (JB and JBl). We included all titles and abstracts screened in by any reviewer. Two reviewers (JB and JBl) independently screened full texts, resolving disagreements by discussion or reference to a third author (RF). Consistent with scoping review methodology, we did not exclude papers on the basis of poor methodology as we aimed to describe and summarise currently available evidence (Arksey & O’Malley, 2005; Tricco et al., 2018).

### Data extraction

We extracted and tabulated data on the following: first author and title; year of publication; country of study; study objectives; population and sample size; evaluation design (Eccles et al., 2003); intervention type (Grimshaw et al., 2012); outcomes; and key results or conclusions reported by the authors. Two reviewers (JB and RF) piloted full text data extraction before two reviewers (JB and JBI) independently extracted data, resolving any disagreements by discussion or reference to a third author (RF).

**Fig. 1:**
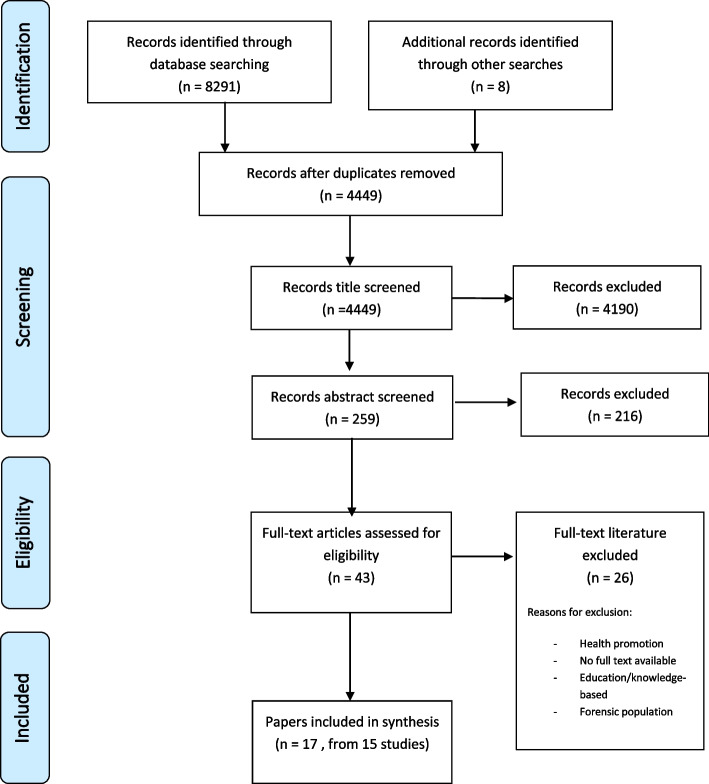
PRISMA flow diagram

Figure 1 demonstrates the search strategy and screening process in a PRISMA flow diagram.

Table 1 displays the 15 studies (17 papers) included in data synthesis with full extraction data.

**Table 1:** Summary of included studies

**First ** **author and year of publication**	**Country**	**Implementation aim**	**Study population**	**Study design**	**Implementation intervention**	**Outcomes**	**Author conclusions**
Arif, T. 2018	UK	To improve the uptake of blood borne virus screening and vaccination	6452 adult males in one category B prison	Uncontrolled before and after	Promotional campaign with printed materials and education for incarcerated persons Rapid hepatitis vaccination schedule and testing offered to all current and new incarcerated persons	Compliance to guidelines Vaccination and screening uptake	Increased rate of hepatitis B and hepatitis C vaccinations offered. Prison guard understaffing judged to affect uptake of services.
Beyda, R. M. 2018	USA	To improve contraception counselling and initiation in young adolescent women	306 juvenile female detainees in pre-adjudicational centre	Uncontrolled before and after	Modified intake form to collect information on contraception Staff training on contraception counselling Printed materials for patients	Proportion of women initiated on contraception Proportion of women continuing contraception use	Higher proportions of women counselled, initiated and continuing to use contraception.
Cabelguenne, D. 2018 and Lerat, M. C. 2011	France	To determine psychiatrists and pharmacist’s adherence to prescribing guidelines	1249 adult males in one prison	Uncontrolled before and after	Collaborative guideline development between psychiatrists and pharmacists	Mean daily dose of benzodiazepine prescribed	Sustained reduction in dosage over the 15 years, although no reductions in those already taking smaller doses. Reduced number of patients on benzodiazepines. Introduction of non-pharmacological treatment during follow up may have contributed to dose reductions.
Elwood Martin, R. 2004	Canada	To determine impact of implementation of a nurse-led cervical screening clinic	484 adult females in one correctional centre	Uncontrolled before and after	Development of a nurse-led cervical screening clinic providing one-to-one education, screening and discussion around treatment	Proportion of women screened and tested	No change in proportion of women screened after intervention. Women over age of 30 years were less likely to have a smear whilst those with lower education status and longer sentences were more likely to have a smear.
Emerson, A. M. 2020	USA	To describe the 1-year postintervention rates of sexual health education on cervical health literacy and screening	133 adult females in two prisons	Mixed methods study, including uncontrolled before and after	Implementation of sexual health empowerment protocol with group education sessions	Cervical cancer screening knowledge, beliefs, self-efficacy and confidence Cervical cancer screening history and health history	Increased number of women with up-to-date screening. Higher cervical health literacy scores.
Finnie, A. J. 2018	UK	To increase referrals to health trainers from clinicians and screening clinics	Unspecified number of males in one category C prison	Uncontrolled before and after	Alerts created on electronic health record for patients eligible and due health checks Referral tool updates with further alert tools Employment of local champions	Number of referrals to health trainers	Increased referrals, with consistent improvement over each intervention cycle. Operational disruption appeared to reduce referrals for three months.
Francis-Graham, S. 2020	UK	To determine effectiveness of a pathway of care for hepatitis C screening and treatment in prison	12964 adult males in one category B prison	Uncontrolled before and after	Implementation of a hepatitis C testing pathway Introduction of referrals direct to multidisciplinary team	Proportion of testing offered and accepted Proportion of referrals to multidisciplinary team	Difficulty engaging prison staff in testing pathway due to staffing pressures. Increased testing following adaptation of pathway, which remained below national targets.
Lee, T. 2016	USA	To assess the impact of implementing psychiatric practice guidelines on medication costs and youth aggression in a juvenile justice facility	Three juvenile justice facilities	Controlled interrupted time series analysis	Implementation of psychiatric practice guidelines	Medication cost by proxy of medication use Mental health acuity scores Aggression-related incident rates	Reduction in psychiatric medication costs over 10 years at the intervention facility compared to increases at control facilities. Reduced aggression-related incidents reported.
Lin, C. H. 2019	USA	To improve diabetes management of detained persons through a pharmacist-led diabetes clinic	240 adult males from two jail facilities	Uncontrolled before and after	Implementation of a pharmacist-led diabetes clinic	Glycaemic control (HbA1c) Proportion prescribed appropriate statin	Reduced mean HbA1c levels, especially in those with earlier higher HbA1c levels, but increase in those with earlier better control. Increased appropriate statin prescribing.
Meine, K. 2018	USA	To improve perinatal depression screening and management	101 adult females housed at two jail facilities.	Mixed methods study, including uncontrolled before and after	Four improvement cycles including staff engagement, patient engagement, implementation of a perinatal depression screening tool, and referral and treatment tools.	Adherence to protocol Patient semi-structured interviews Screening proportions	Increased proportion of women screened and treated for perinatal depression, although unable to attribute this to any intervention component over the four cycles.
Morey, S. 2019	UK	To improve blood borne virus testing and treatment rates	4280 adult males in two prisons	Uncontrolled before and after	Development of testing pathway offering universal opt-out dry blood spot testing (DBST) Telemedicine (consultant-led) and in-reach nurse-led clinic	Proportion of DBST offered and undertaken Proportion of positives and referrals to treatment team	Increased offers and acceptance of DBST, sustained over six months. Improved attendance rates at telemedicine clinic compared to face-to-face clinics.
O’Toole, S. 2018	Ireland	To explore efficacy of an exercise referral scheme for promoting mental health	30 adult males in one prison	Mixed methods study, including uncontrolled before and after	Exercise referral scheme	Depression and anxiety scale, anger scale and self-esteem symptom scale Patient semi-structured interviews	Improved symptoms scores for anxiety, depression, stress, anger and self-esteem
Pearson, F. 2014 and Pankow, J. 2018	USA	Investigate if a model of HIV care would improve quality of care	14 clusters of matched prisons	Cluster randomised controlled trial	Introduced a local change team with a QI protocol Staff training on protocol	Screening uptake for HIV Proportion of positive HIV cases Patient knowledge surveys Protocol adherence	Improvement in patient knowledge scores and proportion of HIV screening uptake. Reported adequate adherence to the structural components of the improvement protocol in 12 sites.
Reeves, R. 2012	USA	To reduce benzodiazepine and quetiapine prescribing for insomnia	Prisons served by psychiatrists from one state	Uncontrolled before and after	Introduction of prescribing guideline Staff training Allowed for psychiatrists to anonymously compare their prescribing to others’	Proportion of patients prescribed benzodiazepines and quetiapine	Reduced benzodiazepine and quetiapine prescribing.
Toledanes, Y. D. 2021	USA	To evaluate the impact of protocols to improve identification and management of asthma in juvenile detainees	764 juvenile detainees at two facilities	Uncontrolled before and after	Implementation of asthma diagnosis protocol Staff training Printed materials to staff	Prevalence of asthma Proportion of inhaler use Staff adherence to protocol	Reduced recorded prevalence of asthma. Reduction in inhaler costs.

## Results

### Selected studies

Our searches yielded 4449 citations, out of which we screened 259 abstracts and then 43 full texts to include 15 studies (17 papers; Fig. 1). The studies were published between 2004 and 2021.

We found one randomised controlled trial (Pankow et al., 2018; Pearson et al., 2014) and one controlled interrupted time series analysis (Lee et al., 2016). The other 13 studies employed uncontrolled before and after designs, three of which were included within mixed-methods studies (Emerson et al., 2020; Meine, 2018; O’Toole et al., 2018). Table 1. summarises features of each study.

Eight studies took place in US detention centres (Beyda et al., 2018; Emerson et al., 2020; Lee et al., 2016; Lin et al., 2019; Meine, 2018; Pankow et al., 2018; Pearson et al., 2014; Reeves, 2012; Toledanes et al., 2021), four in the UK (Arif, 2018; Finnie, 2018; Francis-Graham et al., 2020; Morey et al., 2019), and one each in France (Cabelguenne et al., 2018; Lerat et al., 2011), Ireland (O’Toole et al., 2018) and Canada (Elwood Martin et al., 2004).

Twelve studies involved adult custodial settings; those holding males exclusively in seven studies (Arif, 2018; Cabelguenne et al., 2018; Finnie, 2018; Francis-Graham et al., 2020; Lerat et al., 2011; Lin et al., 2019; Morey et al., 2019; O’Toole et al., 2018) and holding females exclusively in three (Elwood Martin et al., 2004; Emerson et al., 2020; Meine, 2018). Two studies did not specify gender of the incarcerated persons (Pankow et al., 2018; Pearson et al., 2014; Reeves, 2012). Three studies were conducted in custodial settings for juveniles, with two housing both male and female juveniles (Lee et al., 2016; Toledanes et al., 2021) and one exclusively female setting (Beyda et al., 2018).

Of the eight US studies, three occurred in jails (Emerson et al., 2020; Lin et al., 2019; Meine, 2018), three in juvenile detention facilities (Beyda et al., 2018; Lee et al., 2016; Toledanes et al., 2021), one in prison (Reeves, 2012), and one in paired prisons or jails (Pankow et al., 2018; Pearson et al., 2014). Of the four UK studies, one studied a Category A (high security) prison (Francis-Graham et al., 2020), two studied Category B (remand and long-term) prisons (Arif, 2018; Morey et al., 2019) and once studied a Category C (training and resettlement) prison (Finnie, 2018).

### Types of implementation intervention

We grouped interventions broadly into professional behaviour change and patient education; 12 studies evaluated interventions that concentrated on professional behaviour change (Beyda et al., 2018; Cabelguenne et al., 2018; Elwood Martin et al., 2004; Finnie, 2018; Francis-Graham et al., 2020; Lee et al., 2016; Lerat et al., 2011; Lin et al., 2019; Meine, 2018; Morey et al., 2019; Pankow et al., 2018; Pearson et al., 2014; Reeves, 2012; Toledanes et al., 2021) and three evaluated patient-mediated interventions involving educating or empowering patients (Arif, 2018; Emerson et al., 2020; O’Toole et al., 2018).

Ten studies evaluated multifaceted strategies which combined interventions (Arif, 2018; Beyda et al., 2018; Cabelguenne et al., 2018; Finnie, 2018; Francis-Graham et al., 2020; Lerat et al., 2011; Meine, 2018; Morey et al., 2019; Pankow et al., 2018; Pearson et al., 2014; Reeves, 2012; Toledanes et al., 2021). Eight studies evaluated educational meetings, largely aiming to improve staff knowledge and patient health literacy. For example, Elwood Martin et al. (2004) evaluated one-to-one nurse-led education sessions explaining the need for cervical cancer screening. Three study interventions drew on local opinion leaders, defined elsewhere as “individuals perceived as credible and trustworthy, who disseminate and implement best evidence” (Flodgren et al., 2019). For example, Pearson et al. appointed “local change teams” (Pankow et al., 2018; Pearson et al., 2014) led by senior healthcare staff with advanced training who acted as educators for the rest of their teams. Two studies evaluated printed educational materials. For example, Beyda et al. (2018) included leaflets written for patients providing detailed information on contraception options. System alerts were evaluated in two studies. For example, Finnie (2018) included prompts in electronic health records to identify patients due for health checks.

### Targeted healthcare conditions

Four studies targeted the prevention and management of communicable diseases, specifically hepatitis B (Arif, 2018), hepatitis C (Arif, 2018; Francis-Graham et al., 2020; Morey et al., 2019) and human immunodeficiency viruses (HIV) (Pankow et al., 2018; Pearson et al., 2014). Four studies concerned mental health, specifically perinatal depression (Meine, 2018) and antipsychotic prescribing (Cabelguenne et al., 2018; Lee et al., 2016; Lerat et al., 2011; Reeves, 2012). Four studies targeted screening programmes or health promotion, specifically cervical cancer screening (Elwood Martin et al., 2004; Emerson et al., 2020), health checks (Finnie, 2018) and exercise (O’Toole et al., 2018). Two studies targeted long term conditions, asthma (Toledanes et al., 2021) and diabetes (Lin et al., 2019). One study targeted contraception (Beyda et al., 2018).

### Outcomes

The most commonly reported outcomes were processes of care, with 13 studies reporting testing, prescribing and referrals (Arif, 2018; Beyda et al., 2018; Cabelguenne et al., 2018; Elwood Martin et al., 2004; Emerson et al., 2020; Finnie, 2018; Francis-Graham et al., 2020; Lee et al., 2016; Lerat et al., 2011; Lin et al., 2019; Meine, 2018; Morey et al., 2019; Pankow et al., 2018; Pearson et al., 2014; Reeves, 2012). The majority of these focused upon screening uptake (seven studies) (Arif, 2018; Elwood Martin et al., 2004; Emerson et al., 2020; Francis-Graham et al., 2020; Meine, 2018; Morey et al., 2019; Pearson et al., 2014). Three studies used patient outcomes such as glycaemic control or symptom scores (Lin et al., 2019; O’Toole et al., 2018; Toledanes et al., 2021). One study assessed patient knowledge (Emerson et al., 2020).

### Author conclusions

All studies bar one (Elwood Martin et al., 2004) reported positive impacts of interventions. For example, there was a statistically significant decrease in the prevalence (and likely overdiagnosis) of asthma in juvenile detainees at two facilities, falling from 18.2% to 11.2% following the implementation of an asthma diagnosis protocol (*p* < 0.0001) (Toledanes et al., 2021). A cluster randomised controlled reported that addition of a protocol-based approach to HIV care doubled the odds of successful delivery of HIV prevention, screening and linkage to treatment (Pearson et al., 2014). The success of this strategy was attributed to high adherence by prison staff to the improvement strategy processes (Pankow et al., 2018).

## Discussion

Considering the significant healthcare needs and vulnerability of the incarcerated population, our scoping review found relatively few evaluations of strategies to improve the uptake of evidence-based healthcare. Even amongst those evaluations identified, only two used rigorous study designs. Therefore, any drives to improve care will either depend on a weak evidence base or need to draw upon rigorous evidence generated in settings that may not be generalisable to prisons.

The majority of studies used uncontrolled before and after designs and reported improvements in care. Such designs are prone to major biases, such as maturation effects, when the passage of time brings about changes in the study units independent of the intervention, or regression to the mean, if study units selected on the basis of low performance subsequently tend to give scores closer to the average (Eccles et al., 2003; Goodacre, 2015). For example, Lin et al. (2019) reported a reduction in mean HbA1c outcomes after introducing pharmacist-led diabetes clinics. This reduction was mostly observed in individuals with higher pre-intervention HbA1c levels and hence this apparent improvement could be explained by regression to the mean rather than a true intervention effect. Furthermore, most studies took place in either a single facility or a small number of sites housing incarcerated populations, which may be self-selected and potentially more amenable to implementation interventions. Such selection bias would limit generalisability.

Most evidence was from US settings, which given differing terminology and criminal justice systems, may not be generalisable to other settings. For example, in the US, the term ‘prison’ refers to a long-term facility owned by either a state or the federal government housing those convicted of serious crimes. In contrast, in the UK for example, the term ‘prison’ refers to a facility holding long- and short-term incarcerated people, including those awaiting trial. Therefore, in a UK setting, a single site may hold incarcerated persons of varying sentence lengths compared to separation of those on remand in a US setting.

Defining and describing interventions was problematic given a lack of standardised descriptive terminology. Our grouping was based upon an existing taxonomy (Grimshaw et al., 2012), which may not have captured nuanced aspects of the interventions we identified. Similarly, it would be difficult to draw generalisable conclusions about intervention effectiveness from the evaluations of multiple cycles of varying interventions and multifaceted interventions. Together, these limitations in the literature pose problems for those looking to adopt or adapt evaluated interventions given uncertainties about their precise characteristics. For example, Reeves (2012) concluded that education, in combination with guideline amendment and peer profiling, was successful in achieving lasting changes in benzodiazepine prescribing. However, the educational intervention was mentioned several times without elaboration of its content. There are many different ways of delivering education with varying success and so the lack of common language and detail provides sparse information for those planning similar approaches. We also observed that the majority of studies relied upon education, which may have limited sustainability.

The conditions targeted largely reflect the recognised priorities for incarcerated populations of communicable diseases and mental health. Blood borne viral infections, substance misuse, depression and post-traumatic stress disorder are all highly prevalent in incarcerated populations (Kinner & Young, 2018). However, relatively few studies targeted other common long-term conditions typically managed in primary care, such as hypertension or asthma, as well as conditions associated with aging populations, such as atrial fibrillation and dementia. These conditions are often amenable to treatments or management strategies that can improve quality of life and longevity. For incarcerated people awaiting trial or serving shorter sentences, access to prison healthcare services offers opportunities for care for those with poor or inconsistent engagement with community primary care. Although men typically account for the majority of the incarcerated population (Walmsley, 2017), we noted that few studies focused on women’s healthcare needs, which may be greater (Public Health England, 2018); recent research has found that incarcerated females are more likely than their male counterparts to suffer from long-term physical health conditions (Wright et al., 2021) and experience mental health problems (Tyler et al., 2019). Indeed, self-harm rates have been found to be over ten times higher in women than for men in prison (Hawton et al., 2014).

Most outcomes concerned processes of care, some of which were evidence-based. For example, Reeves (2012) aimed to reduce prescribing recognised as causing potential patient harm. However, the utility of other outcome measures was sometimes uncertain, such as numbers of referrals (Finnie, 2018). Studies reporting outcomes such as symptom scores, as seen in O’Toole et al. (2018), provide more direct information relevant to patients but are prone to reporting bias due to the nature of self-reporting and subjective scales (Higgins et al., 2021). Whilst our review focused on measurable outcomes, we recognise the importance of outcomes which are less amenable to measurement, especially through routinely collected data, such as patient experience and autonomy.

### Study strengths and limitations

Our study was novel in aiming to identify and describe evaluations of implementation interventions in the prison setting. We followed widely recognised methods for scoping reviews, including a reasonably comprehensive search strategy (Arksey & O’Malley, 2005). Apart from the limited quality of the evaluations we identified, we acknowledge three main limitations of our methods. First, our scoping review did not exclude on the basis of study quality. However, we noted the preponderance of weak designs with low validity for causal inference. Second, we are uncertain of the extent of publication bias and evaluations with favourable findings could be more likely to be reported than those showing no benefit. Third, we focused our review on studies assessing the effectiveness of implementation strategies and acknowledge that further valuable insights into why strategies succeed or not could be added by mixed-method process evaluations (Grant et al., 2013).

### Implications for policy and research

Our findings mean that policymakers have little empirical basis for selecting and applying interventions to improve the uptake of evidence-based health care in prisons. There is a growing body of evidence from randomised trials and rigorous quasi-experiments for various implementation interventions in other healthcare settings, for example 140 studies evaluating the effects of audit and feedback (Ivers et al., 2012) and 108 studies evaluating the effects of computerised clinical decision support systems (Kwan et al., 2020), yet none of these studies concerned incarcerated populations. Whilst the findings of such systematic reviews could be applied with a degree of judgment (Sackett et al., 1996), prison settings present unique challenges to implementation (such as system and resource constraints and high health needs) which undermine generalisability of the wider evidence base. We did identify one robustly designed study, which demonstrates the feasibility of implementation trials in a prison setting and which found that quality improvement involving defined leadership, local change teams and staff training improved the uptake of HIV screening (Pankow et al., 2018; Pearson et al., 2014).

Our study holds up a mirror to the prison healthcare policy and research field. There have been calls for equivalence of healthcare and outcomes between incarcerated and community populations (Charles & Draper, 2012). The lack of rigorous evaluations we found suggests the need to re-balance research resources and efforts to start building a stronger evidence base to address the gaps between recommended and actual care in prisons. This will require capacity-building in this field of research, as well as collaborative work to allow secure data-sharing between prison healthcare providers and researchers. This would, for example, allow the use of routinely collected data as outcomes in future randomised trials of implementation strategies (Wolfenden et al., 2021).

## Conclusion

There is a paucity of high-quality evidence on the effectiveness of strategies to improve the implementation of evidence-based health care in prisons. Whilst evidence from other settings may still be relevant, it is unlikely to take account of the highly challenging context of prison healthcare and the substantial needs of the incarcerated population. There is a case for more concerted efforts to develop and evaluate implementation interventions using rigorous evaluation designs.

## Supplementary Information

**Additional file 1.** Scoping Reviews (PRISMA-ScR) Checklist. This file contains details of the Preferred Reporting Items for Systematic reviews and Meta-Analyses extension for Scoping Reviews (PRISMA-ScR) Checklist (Tricco et al., 2018).

**Additional file 2.** Search Strategy. This file contains details of the complete search strategy performed.

## Data Availability

The main text and appendices include all relevant data from this study.

## Abbreviations

BBV: Blood borne virus
DBST: Dry blood spot testing
HbA1c: Glycated haemoglobin
HIV: Human immunodeficiency virus
QI: Quality improvement
UK: United Kingdom
US: United States of America
