# Supplementary material for: Interventions to improve the implementation of evidence-based healthcare in prisons: a scoping review
Source: Health Justice. 2023 Jan 3;11:1. doi: 10.1186/s40352-022-00200-x (PMC9809036; doi:10.1186/s40352-022-00200-x)
Supplement: Supplementary file 2 — Additional file 2. Search Strategy. This file contains details of the complete search strategy performed. [file 40352_2022_200_MOESM2_ESM.docx]

# Appendix 2: Search strategy

**EMBASE and Medline**

| 1. Prisons/ |  |
| --- | --- |
| 2. Prisoners/ |  |
| 3. (Jail* or penitent* or Gaol* or reformatory).tw. |  |
| 4. ((penal or secure or correctional) adj (institution* or system* or unit* or facilit* or centre* or center*)).tw. |  |
| 5. (Incarceration or imprisonment or detention).tw. |  |
| 6. (Inmate* or Offender* or Prison* or Convict* or detainee* or re-offend* or reoffend* or incarcerat* or felon*).tw. |  |
| 7. or/1-6 |  |
| 8. Evidence-based/ or evidence based/ or evidence-based practice/ or evidence-based medicine/ |  |
| 9. (evidenced-based or evidenced based).tw. |  |
| 10. EBP*.tw. |  |
| 11. Evidence-informed.tw. |  |
| 12. Best practice/ |  |
| 13. Patient-centred care/ |  |
| 14. (Patient adj (centered or centred)).tw. |  |
| 15. (Patient-centered or patient-centred).tw. |  |
| 16. (QOF or quality outcomes framework).tw. |  |
| 17. (guideline adj (adherence or implementation or promotion)).tw. |  |
| 18. Implementation science/ |  |
| 19. (implementation adj2 (guideline or best practice or evidence-based practice)).tw. |  |
| 20. Quality improvement/ |  |
| 21. health promotion/ |  |
| 22. or/8-21 |  |
| 23. 7 and 22 |  |

**CINAHL**

**
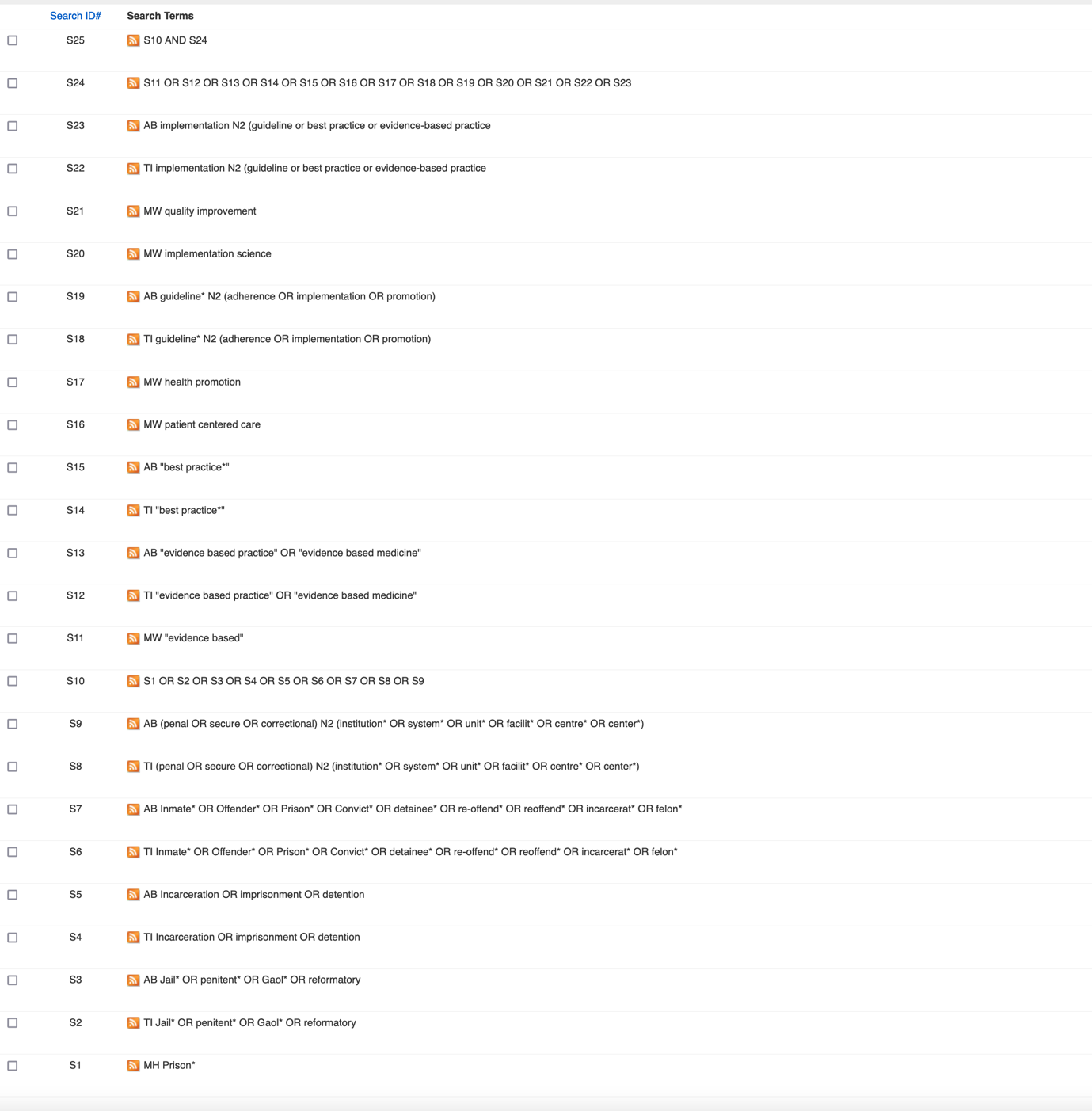
**

**Scopus**

( ( TITLE-ABS-KEY ( prison* OR jail* OR penitent* OR gaol* OR reformatory ) OR TITLE-ABS-KEY ( ( penal OR secure OR correctional ) W/1 ( institution* OR system* OR unit* OR facilit* OR centre* OR center* ) ) OR TITLE-ABS-KEY ( incarceration OR imprisonment OR detention ) OR TITLE-ABS-KEY ( inmate* OR offender* OR prisoner* OR convict* OR detainee* OR re-offend* OR reoffend* OR incarcerat* OR felon* ) ) ) AND ( ( TITLE-ABS-KEY ( [evidence-based] ) OR TITLE-ABS-KEY ( [evidence based] ) OR TITLE-ABS-KEY ( [Quality Improvement] ) OR TITLE-ABS-KEY ( [Implementation science] ) OR TITLE-ABS-KEY ( [health promotion] ) OR TITLE-ABS-KEY ( [best practice*] ) ) )

**Web of Science**

**
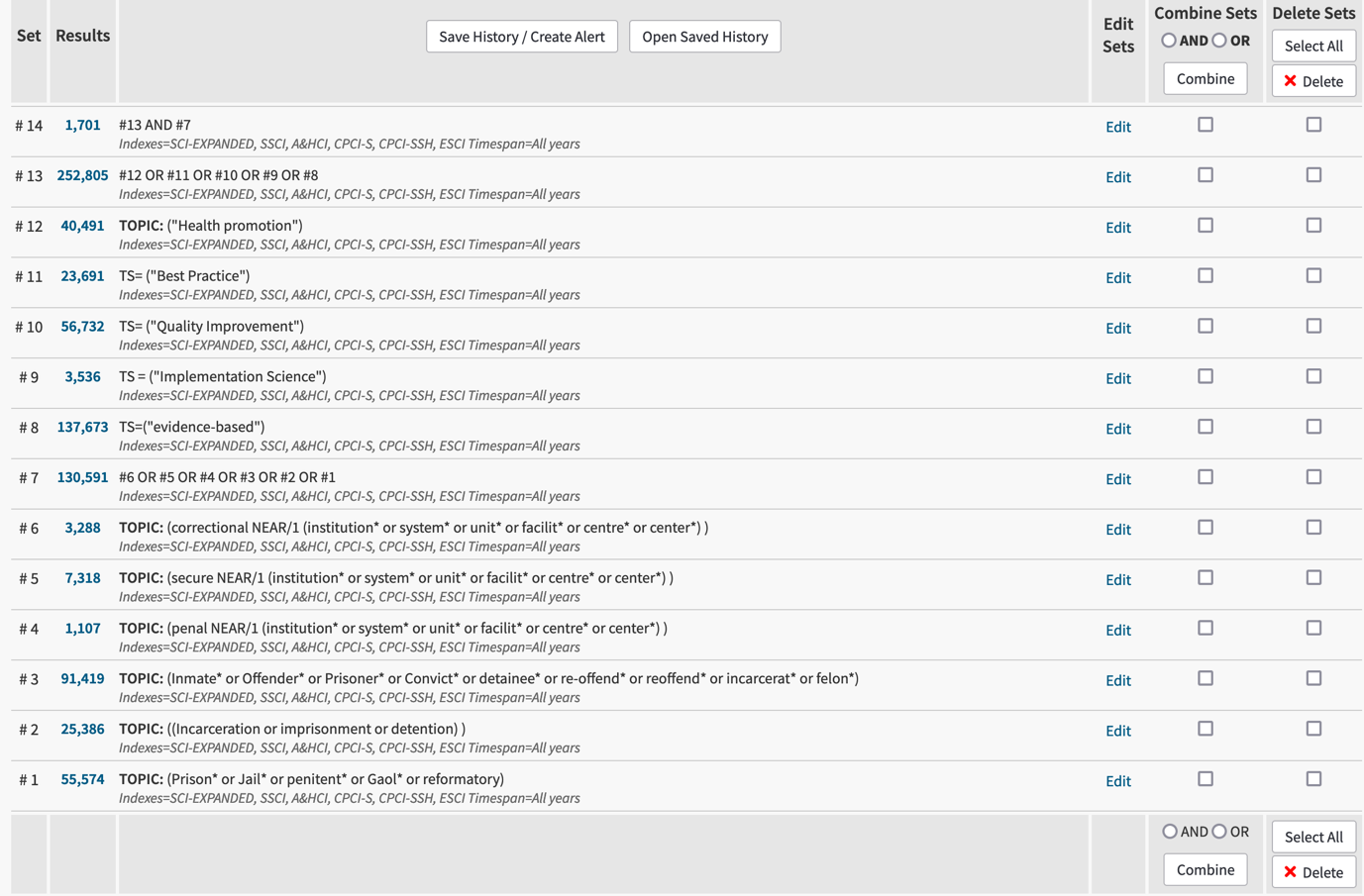
**
